# Graft versus host disease diagnosis through biopsy of the oral mucosa lesions

**DOI:** 10.1590/S1808-86942010000300023

**Published:** 2015-10-20

**Authors:** Alexandre Pinto Maia, Pollianna Muniz Alves, Roberto Tiago Alves Pinheiro, Kátia Maria Gonçalves Marques, Hébel Cavalcanti Galvão

**Affiliations:** 1 MSc. In Periodontics/ UFRN, PhD student in Oral Pathology/UFRN, CAPES scholarship holder; 2 MSc. In Oral Diagnosis/UFPB, PhD student in Oral Pathology/UFRN – CAPES scholarship holder; 3 DDS. Resident in Maxillo-Facial Surgery – Hospital da Restauradlo, Recife, PE; 4 DDS – HEMOPE, Recife/PE; 5 PhD in Oral Pathology, Professor at the Graduate Program in Oral Pathology/ UFRN

**Keywords:** biopsy, graft vs host disease, mouth mucosa

## INTRODUCTION

Bone marrow transplant (BMT) is a treatment modality which brings about a chance of cure or a better prognosis for patients with malignant or benign blood diseases, such as thalassemia and sickle cell anemia, besides tumors such as renal cell carcinoma^1^, ^2^.

Among BMT complications, the main one is the Graft Versus Host Disease (GVHD), which is an immune reaction resulting from the grafting of immunocompetent cells from a donor to an immunocompromised host. GVHD happens in between 50% and 80% of the patients submitted to BMT, and it is the main cause of long term morbi-morbidity^3^.

Although the pathophysiology of GVHD is not fully unveiled, it is believed that it is primarily mediated by T-cells recognizing the tissues of the patient as antigens because of histocompatibility differences^4^, ^5^.

GVHD affect more frequently the skin, liver, gastrointestinal tract and oral mucosa6. The oral manifestations of GVHD include lichenoid lesions, mucosa ulcerations, atrophic glossitis, diffuse erythema, taste disorders and salivary glands hypofunction causing xerostomia^3^, ^4^.

Having said that, the present paper aims at reporting on a case diagnosed as GVHD, based on a biopsy of the oral mucosa, as well as discussing the relevant aspects of this pathology.

## CASE PRESENTATION

Male, 15 year old, came to our dental clinic approximately 30 days after noticing whitish and diffuse lesions on his mouth. He reported he was not a smoker nor drank alcoholic beverages. The patient described having had severe aplastic anemia, and having been submitted to BMT approximately two years in the past.

**Figure 1:**
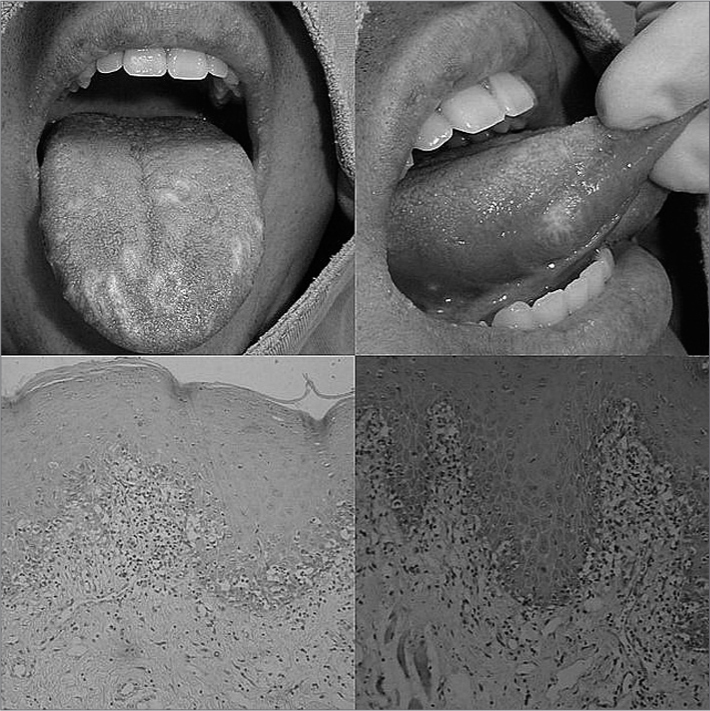
Upper part – diffuse whitish lesions on the tongue dorsum and lateral border. Lower part: one can notice parakeratin, blurring of the basal layer border and predominantly lymphocytic inflammatory infiltrate located on the epithelial junction area in a band shape (H/E, 10x e 40X, respectively).

Examining his mouth we noticed painless whitish lichenoid lesions on the mouth mucosa, palate and tongue (Figure 1). There were no suggestive lesions of such disorder on the skin nor in other organs, renal and liver functions were normal and he had a normal chest x-ray as well.

The onset of the lesions coincided with the suspension of cyclosporine – immunosuppressant, used to avoid de development of GVHD. Since the patient had been submitted to a BMT before and the clinical findings of a whitish lichenoid lesion matched descriptions of GVHD, we did a core biopsy of the tongue.

Histologically we could notice the presence of an oral mucosa fragment coated by hyperkeratinized squamous stratified epithelium, with areas of blurring on the basal layer. The underlying connective tissue was dense and fibrous, with intense inflammatory infiltrate, predominantly lymphocytic, located on the junction portion in a band shape (Figure 1). Therefore, by associating the previous clinical history with these histopathological findings we confirm the diagnosis of GVHD.

Cyclosporine was reinstated in a low dose, associated with rinsing the mouth with Betamethasone 0.1mg/ml, TID for 15 days, changing to once a day for an undetermined amount of time. The patient is being followed up, without recurrences.

## DISCUSSION

GVHD can be limited, affecting only the oral mucosa or the skin, or it can be spread, affecting two or more organs^5^, ^6^. The case hereby reported is the limited form of the GVHD, in which only the oral mucosa was affected, without any other clinical manifestation being observed.

The lichenoid lesions are the most commonly seen, and are distributed along the jugal, gengival and lingual mucosas. The differential diagnosis includes lichen planus, multiform erythema, lupus erythematous and lesions associated with chemotherapy^1^, ^2^, ^3^, ^4^.

The lesions seen in the oral mucosa of this patient had a lichenoid aspect, and we ruled out the possibility of the patient having an autoimmune disease – according to his interview, as well as having chemotherapy-related lesions because of the time interval between it and the lesions appearing – which was 2 years.

Therefore, considering his past of BMT and the histopathology findings, the conclusive diagnosis was of GVHD.

## FINAL REMARKS

Because of the high incidence of oral mucosa involvement and the ease with which the biopsy can be made, such procedure can be used aiming at the definitive diagnosis of GVHD in post-BMT patients, thus contributing to a better prognosis and enhanced quality of life for these patients.
